# A case report of IgG4-RD involving bilateral renal pelvis and literature review

**DOI:** 10.1097/MD.0000000000043453

**Published:** 2025-08-15

**Authors:** Shaolong Zhang, Chuanjian Chen, Chunhao Mo, Hui Ding

**Affiliations:** a Department of Urology, Gansu Province Clinical Research Center for urinary system disease, Lanzhou University Second Hospital, Lanzhou, China.

**Keywords:** case report, IgG4 related disease, renal pelvis masses

## Abstract

**Rationale:**

IgG4 related disease (IgG4-RD) is a systemic, and immune mediated chronic disease. IgG4-RD rarely presents as a bilateral renal pelvis soft tissue mass, which may be misdiagnosed as malignant urothelial tumor, leading to invasive surgical intervention and organ damage. Through the introduction of this rare case, the understanding of IgG-RD by clinicians can be increased, so as to reduce the misdiagnosis of tumor with space-occupying lesions in clinical practice, save medical resources, and reduce the unnecessary surgical burden of patients.

**Patient concerns:**

A 70-year-old male patient presented to our hospital with the chief complaint of “occupying bilateral renal lesions found on physical examination for 3 months.” No visual hematuria, back pain or fever, no discomfort such as frequent, urgent or painful urination. Hypertension for 3 years, which is controlled by oral medication. No positive signs on physical examination.

**Diagnosis:**

IgG4-RD involving bilateral renal pelvis.

**Intervention:**

Computed tomography scan and magnetic resonance imaging incidentally discovered a mass located in the bilateral renal pelvis. The radiological examination results highly suggest malignant tumors. Therefore, the patient underwent right laparoscopic biopsy and the results showed that fibrous tissue hyperplasia with extensive plasma cell infiltration and lymphoid follicle formation. Immunohistochemical staining showed IgG4 positive plasma cells > 40/HPF, plasma cells IgG4/ IgG > 40%. The patient was treated with glucocorticoids in combination with immunosuppressive drugs.

**Outcomes:**

Erythrocyte sedimentation rate and IgG4 returned to normal after first-line drugs treatment. The size of bilateral masses has been significantly decreased. The patient’s kidney function is within the normal range.

**Lessons:**

In patients with bilateral renal pelvis masses, IgG4-RD should be considered in differential diagnosis to avoid unnecessary surgical intervention, and serum IgG4 level is the important biomarker. Glucocorticoids, immunosuppressive drugs, and biological agents are the first-line therapeutic medication for IgG4-RD.

## 1. Introduction

**Figure 1.**
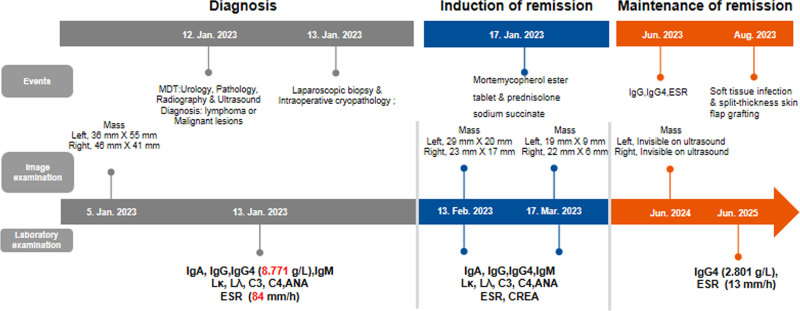
Timeline of diagnosis and treatment for this case.

IgG4 related disease (IgG4-RD) is a systemic, and immune mediated chronic disease.^[1]^ This disease can involve various organs systems, such as the pancreas, biliary gland, thyroid, lung, kidney, retroperitoneum and gastrointestinal.^[2–4]^ IgG4-RD is characterized by elevated serum IgG4 levels and IgG4-positive plasma cell infiltration in affected tissues, and abundant fibrosis. IgG4-RD was described in 2003, and it typically affects over 50 years old people with a male to female ratio that ranges from 1.6:1 to 4:1.^[1,5]^ The disease can occur simultaneously in multiple organs, and the digestive tract is one of the most commonly involved organs in IgG4-RD, such as autoimmune pancreatitis and sclerosing cholangitis. However, it is very rare for the disease to first appear in both renal pelvises, and easily misdiagnosed as renal pelvic tumor. This study reports a IgG4-RD case with only involving bilateral renal pelvises. We discuss the disease characteristics and management process of IgG4-RD to raise awareness of the disease (Fig. 1).

## 2. Case presentation

**Figure 2.**
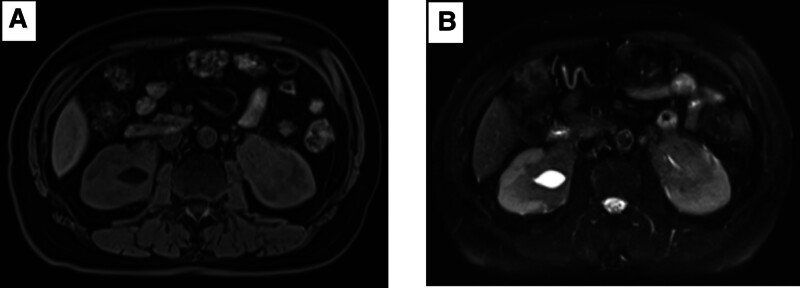
Abdominal MRI imaging showed space-occupying lesions at both renal pelvis.

A 70-year-old male patient presented to our hospital with the chief complaint of “occupying bilateral renal lesions found on physical examination for 3 months.” No visual hematuria, back pain or fever, no discomfort such as frequent, urgent or painful urination. Hypertension for 3 years, which is controlled by oral medication. No positive signs on physical examination. Urine routine test is normal. Blood routine test revealed hemoglobin 119 g/L, total RBC count 3.83 × 10^12^/L. Colour ultrasound of the urinary system showed hypoechoic lesions at the bilateral pelvic-ureteral junction, bilateral hydronephrosis, and bilateral dilatation of the upper ureter. Magnetic resonance imaging (MRI) showed bilateral thickening of the renal pelvis and ureteral wall with local soft tissue density, wrapping the junction of the renal pelvis and pelvis, 46 mm × 41 mm and 36 mm × 55 mm on the right and left sides respectively; enhanced scan showed significant thickening of the renal pelvis and ureteral wall with progressive enhancement and multiple enlarged lymph nodes adjacent to the aorta, considering renal pelvic carcinoma, not excluding lymphoma (Fig. 2). Computed tomography (CT) and PET-CT also showed bilateral soft tissue masses in the renal pelvis, thickening of the upper ureteral wall and a heterogeneous increase in glucose metabolism; consider malignant lesions.

**Figure 3.**
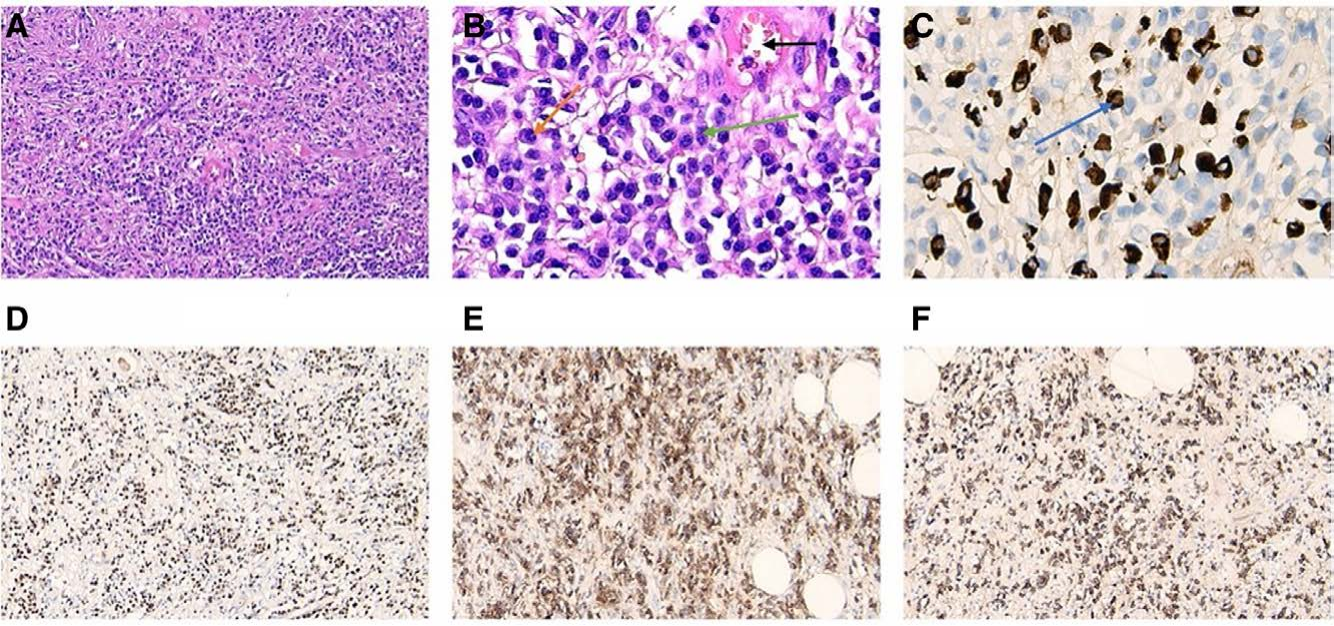
Pathological results. HE staining, 10×, showed a large area of plasma cells, lymphocytes and capillaries (A), HE staining, 40×, black arrows indicate blood vessels, orange arrows indicate plasma cells with nuclei tilted to one side, and green arrows indicate lymphocytes (B), immunohistochemistry, 40×, showed a large number of IgG4 + cells per high power field (C), immunohistochemistry, 10×, showed a large area of MUM1 + cells (D), immunohistochemistry, 10×, showed a large area of CD38 + plasma cells (E), immunohistochemistry, 10×, showed CD138 + plasma cells in the visual field (F). HE = hematoxylin-eosin staining.

**Figure 4.**
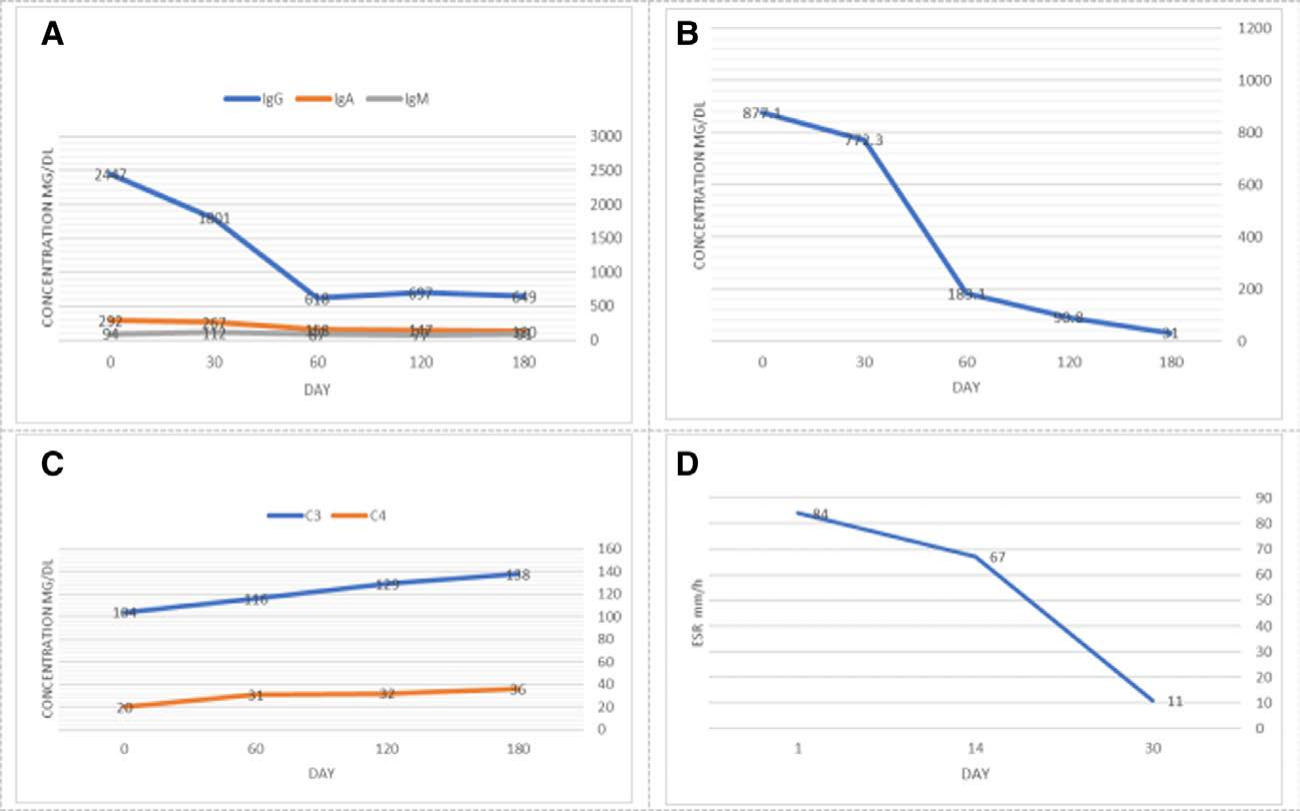
Clinical laboratory results. The concentration of IgG was significantly higher than IgA and IgM, the concentration of IgA, IgG, and IgM at the beginning of treatment, 1 month after treatment, 2 months after treatment and 4 months after treatment, 6 months after treatment (A); changes in the concentration of IgG4 (B); changes in the concentration of C3,4 (C); changes in the erythrocyte sedimentation rate (ESR) (D).

To further clarify the diagnosis, the patient was advised to undergo a puncture biopsy of the lesion tissue. However, due to the proximity of the lesion tissue to the renal artery and ureter, the ultrasonographer declined to puncture it. A laparoscopic biopsy of the lesion tissue was then performed. The postoperative pathology report showed fibrous tissue hyperplasia with extensive plasma cell infiltration and lymphoid follicle formation. Immunohistochemical staining showed IgG4 positive plasma cells > 40/HPF, plasma cells IgG4/ IgG > 40%, B cells CD20 (+), CD79a (+), T cells CD3 (+), Bcl-2 (+), CD21 (+), follicular dendritic network (+), plasma cells CD38 (+), CD138 (+), ki67 germinal center positive cell count 90% (Fig. 3). To further evaluate and improve the accuracy of the diagnosis, immune and inflammation-related tests were performed on this patient and revealed: IgA 2.92 g/L, IgG 24.47 g/L (IgG4 8.771 g/L), IgM 0.94 g/L, Lκ, 6.98 g/L, lλ2.99 g/L, complement C3: 1.04 g/L, complement C4: 0.20 g/L; positive anti-nuclear antibody (ANA); erythrocyte sedimentation rate (ESR) 84 mm/h, C-reactive protein 5.96 mg/L. The combination with serological and immunohistochemical staining, IgG4-associated renal pelvis-ureteral lesions were considered according to the American College of Rheumatology European League Against Rheumatism classification criteria for IgG4-associated disease (2019). The patient was treated with methylprednisolone 80 mg, ivgtt, qd. After 1 week of treatment, the patient was discharged and given mortemycopherol ester tablet (40 mg, po, tid) combined with prednisolone sodium succinate (80 mg, ivgtt, qd). Except for local skin infections, the patient reported no serious side effects. The results of relevant indicators are shown in the chart below: the concentration of IgG was significantly higher than that of IgA and IgM at the beginning of treatment, however, after treatment, the concentration of IgG, especially IgG4, was significantly decreased over time. At the same time, the ESR decreased from 84 mm/h to the normal range. Correspondingly, the level of C3 and C4 gradually increased (Fig. 4). After 1 month, the left mass has been decreased from 46 × 41 mm to 36 × 30 mm, and the right mass has been decreased from 23 × 17 mm to 29 × 20 mm.

## 3. Discussion

### 3.1. IgG4-RD

IgG4-related disease was first officially described in 2010, and the disease is prevalent in middle-aged and elderly individuals.^[5]^ Criteria for diagnosis of this disease are^[6]^ clinical examination showing diffuse/localized swelling or masses in single or multiple organs; hematological examination showing a raised serum IgG4 concentration (>135 mg/dL); and histopathological examination showing marked lymphocyte and plasmacytic infiltration and fibrosis, as well as infiltration of IgG4 + plasma cells at IgG4/IgG cell ratio over 40% and more than ten IgG4 + plasma cells/high power field. If all the above 3 items are fulfilled, the diagnosis is confirmed.

As a systemic immune-mediated fibroinflammatory disorder, the diagnosis and management of IgG4-related disease (IgG4-RD) often necessitate multidisciplinary team (MDT) collaboration, particularly in cases involving rare anatomical involvement (e.g., bilateral renal pelvis lesions, as in this case) or atypical clinical presentations, where the pivotal role of MDT becomes more pronounced. In this case, initial imaging evaluations (ultrasound, CT, MRI) identified bilateral renal pelvis occupying lesions (right: 46 × 41 mm; left: 36 × 55 mm), with radiological features highly suggestive of malignancy (Fig. 2). Consequently, the MDT (comprising urology, radiology, pathology, and rheumatology departments) conducted a joint consultation and opted for laparoscopic biopsy to clarify the pathological nature. Histopathological examination revealed fibrous tissue hyperplasia with extensive plasma cell infiltration and lymphoid follicle formation. Immunohistochemical analysis further confirmed IgG4-positive plasma cell density > 40/HPF and an IgG4/IgG ratio > 40%, corroborated by serological testing (elevated IgG4 level: 8.771 g/L). These findings collectively satisfied the 2019 ACR/EULAR classification criteria for IgG4-RD. The MDT’s collaborative decision-making not only averted unnecessary radical nephroureterectomy but also achieved organ preservation through a precision-guided therapeutic approach (glucocorticoid induction therapy combined with mycophenolate mofetil maintenance), underscoring the MDT’s critical value in reducing misdiagnosis rates and optimizing clinical pathways.

Notably, the systemic propensity of IgG4-RD (e.g., multi-organ involvement) and its heterogeneous pathological mechanisms (e.g., aberrant B/T-cell activation, fibrotic progression) necessitate sustained MDT engagement in long-term patient management. During the maintenance remission phase (6 months post-treatment), this patient developed a lower extremity soft tissue infection requiring split-thickness skin flap grafting, a complication potentially attributable to infection risk escalation from prolonged immunosuppressive therapy. The MDT balanced disease control and infection management by adjusting the immunosuppressive regimen (tapering glucocorticoid dosage and adjunctive anti-infective therapy). At the latest follow-up (June 5, 2025), imaging confirmed complete resolution of bilateral renal pelvis lesions (Fig. 1), with serum IgG4 levels stabilized at 280.1 mg/dL, indicating sustained remission. This trajectory highlights the indispensability of MDT in dynamic risk assessment, complication intervention, and personalized follow-up.

The cause of the disease is not clear, it can involve multiple organs of the body, such as central nervous system, thyroid glands, lungs, bile ducts, liver, gastrointestinal tract, kidneys, prostate, posterior peritoneum, lymph nodes, and skin.^[4,7–12]^ The kidney is the most commonly reported genitourinary organ involvement. It has reported that IgG4-RKD accounts for 7.0% to 24.6% of IgG4-RD abroad.^[13,14]^ According to the ACR/EULAR diagnostic criteria,^[15,16]^ IgG4-related kidney disease is described as follows:

### 3.2. Kidney

Hypocomplementemia pertains to low serum levels of C3, C4, or both.

Renal pelvic wall thickening can be either unilateral or bilateral, usually without severe stenosis or luminal irregularity.

Low-density areas in both renal cortices can be seen only on contrast-enhanced computed tomography and are usually patchy or round-shaped in appearance.

Overall, the diagnosis of IgG4 RKD requires a comprehensive evaluation of the patient’s clinical manifestations, serum IgG4 levels, imaging examinations, and renal pathology.

This case was discovered by chance through a physical examination, and color Doppler ultrasound showed bilateral renal pelvis masses. Further CT and MRI examinations considered bilateral renal pelvis cancer or lymphoma. Due to the absence of obvious underlying diseases in the patient and the absence of cancer cells in urine cytology, as well as the tumor being located in the renal hilum, the risk of puncture biopsy is high. Retroperitoneal laparoscopic biopsy was performed, and postoperative pathology reported IgG4 related kidney disease. Postoperative supplementary examination of IgG4 related kidney disease indicators showed an abnormal increase in IgG4. From this case, it can be seen that due to the enhancement effect of the patient’s lesion imaging similar to that of a tumor, it is highly susceptible to misdiagnosis as a malignant tumor.

When IgG4 RKD presents as a solid tumor, it is commonly misdiagnosed as lymphoma, urothelial carcinoma, or metastatic tumor, and leading to unnecessary nephroureterectomy. The published reports of IgG4-RD mimicking renal pelvic tumor showed that most patients are male, middle-aged and elderly, with the lesion mainly located on the right side and no hematuria.^[17–23]^

Singh et al reported a case of a 64-year-old man with IgG4-RD mimicking upper tract urothelial carcinoma, and they performed a laparoscopic nephroureterectomy.^[17]^ Wang et al also reported a case of IgG4-RD masquerading as renal pelvic cancer. CT, MRI, retrograde pyelography and PET/CT considered renal pelvic cancer.^[18]^ The patient’s serum levels of high-sensitivity C-reactive protein and IgG4 were found to be elevated in laboratory examinations. The patient underwent steroid therapy, her enlarged lymph nodes diminished after 4 months, and high-sensitivity C-reactive protein level had descended to near normal. In the case we reported, ultrasound, CT, and MRI were performed, and imaging indicated bilateral renal pelvis carcinoma. To determine the nature of the lesions, laparoscopic resection of space-occupying lesions was performed. Intraoperative freeze pathology indicated xanthogranuloma, and postoperative pathology indicated IgG4 RKD. Combined with the rheumatology and immunology department, ESR and IgG4 returned to normal after first-line drug treatment. The size of bilateral kidney mass has been significantly decreased.

### 3.3. The pathophysiology of IgG4-RD

**Figure 5.**
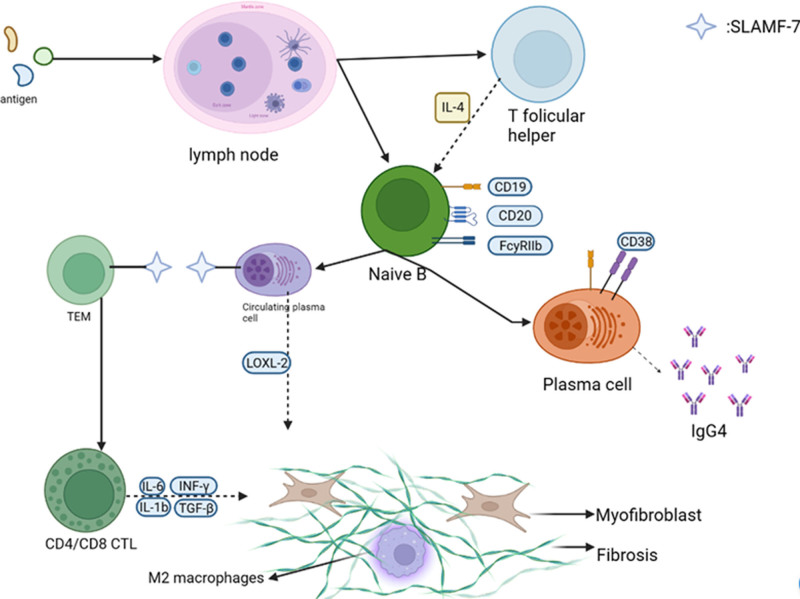
Pathophysiology of IgG4-RD. Antigen stimulation of lymph nodes increased Tfh and native B cells, and the native B cells differentiated into plasma cells, and Tfh cells secreted IL-4 to promote plasma cells to secrete IgG4. Some plasma cells travel to inflammatory tissues and become circulating plasma cells, which activate CD4 and CD8 cytotoxic T cells via SLAMF-7. CD4 and CD8 cytotoxic T cells are important factors in fibrosis. M2 macrophages interact with fibrocytes to further promote tissue fibrosis. IgG4-RD = IgG4 related disease.

The pathophysiology of IgG4-RD includes “inflammatory” phase and “fibrotic” phase.^[24–27]^ The first inflammatory phase of IgG4 RD is characterized by the presence of antigen experienced B and T lymphocytes, which gather at the disease site, participate in antigen-driven interactions that activate each other, and secrete pro-fibrotic molecules such as interleukin-1β, interleukin-6, interferonγ, transforming growth factorβ, platelet derived growth factor B and lysine oxidase homologue 2.^[28–30]^ In particular, M2 macrophages have been shown to infiltrate IgG4 RD lesions and express pro-fibrotic cytokines such as interleukin-10, interleukin-13, interleukin-33, and CCL18,^[31,32]^ which transition the disease from an inflammatory phase to a fibrotic phase. In the fibrosis stage of IgG4 RD, lymphocytes and innate immune cells are replaced by dense stromal reactions, which gradually lead to tissue distortion and organ damage.^[27]^ Although the mechanism associated with the second stage of the disease is still unclear, compared to other immunoglobulin subclasses, it is known that IgG4 antibodies are involved in the resolution of tissue inflammation due to their inherent anti-inflammatory properties(Fig. 5).^[33,34]^

### 3.4. Therapy

#### 3.4.1. Corticosteroids

Due to the recent recognition of IgG4-RD as a systemic disease, comprehensive treatment for its various manifestations is still in the early stages of definition. Corticosteroids are very effective against IgG4-RD, but as they gradually decrease to low doses, they ultimately cannot control inflammation. In addition, clinicians should consider the relapse relief nature of this situation and the potential side effects of glucocorticoids. Moreover, the current literature reported that there are differences in the efficacy and side effects of glucocorticoid therapy due to the different organs involved in IgG4-RD.^[35–37]^

#### 3.4.2. Induction of remission

Although there is no general consensus on the duration of treatment and the tapering regimens, experts suggest that the initial steroid dose should be maintained for at least 2 to 4 weeks, and then gradually reduced by 5 mg every 2 weeks for 3 to 6 months.^[35]^

46% to 90% of patients experience recurrence within 3 years after diagnosis in the same affected organ or different anatomical sites, whether during the reduction period (26%–40% of patients) or after discontinuing glucocorticoid therapy (46%–54% of patients).^[38–41]^

#### 3.4.3. Maintenance of remission

The selection and treatment management of patients who need maintenance treatment are still unclear. Based on expert opinion, patients with multiple organ diseases, elevated serum IgG4 and IgE, and peripheral eosinophilia exhibit the highest risk of recurrence and may benefit from treatment that maintains remission.^[42,43]^

The retrospective studies of AIP patients in Japan showed that the recurrence rate (23%) of patients who continued to receive low-dose glucocorticoid treatment was lower than those who stopped treatment after remission (34%), and the recurrence rate (58%) of patients who stopped low-dose (5 to 7.5 mg daily) steroid treatment after 26 weeks was significantly higher than those who continued maintenance treatment for up to 3 years (23%).^[39,44]^ Nevertheless, it is worth noting that among patients who adhere to the maintenance regimen, patients who receive low-dose prednisone (<5 mg daily) have a higher risk of recurrence.^[45,46]^

### 3.5. Immunosuppressive drugs

#### 3.5.1. Induction of remission

A meta-analysis of 15 observational, non-controlled, and non-randomized clinical trials involving 1169 patients found that patients receiving combination therapy had a higher response rate than those receiving only glucocorticoids (odds ratio 3.36, 95% confidence interval 1.44 to 7.83) or DMARDs (55.31, 13.73 to 222.73) monotherapy.^[47]^ When there are predictive factors for recurrence, such as multiple organ involvement, elevated serum IgG4 and IgE at baseline, and increased peripheral blood eosinophils, disease modified anti rheumatic drugs can be added to first-line steroid therapy to increase the likelihood of achieving disease remission.

#### 3.5.2. Maintenance of remission

Two prospective Chinese clinical trials showed that compared to steroid therapy alone (21% vs 40% and 12% vs 39%, respectively), the addition of mycophenolate mofetil (1–1.5 g/d) or oral cyclophosphamide (50–100 mg/d) to low-dose corticosteroids reduced the recurrence rate after 1 year.^[46,48]^ A meta-analysis of 15 studies involving 1169 patients further confirmed that the combination of glucocorticoids and immunosuppressive drugs was associated with a lower recurrence rate compared to the use of glucocorticoids alone (odds ratios 0.39, 0.20 to 0.80).^[47]^

### 3.6. Biological agents

#### 3.6.1. Induction of remission

Rituximab was the first targeted therapeutic agent for IgG4 RD patients and the most widely used biological agent in this disease.^[49]^ Data from non-controlled, non-randomized prospective and retrospective studies suggest that rituximab leads to disease remission in 67% to 83% of cases, allowing for early reduction in glucocorticoid therapy.^[50–53]^

In a few reports, rituximab is effective at intervals of 15 days for 2 doses of 1 g (rheumatic regimen) or 4 doses of 375 mg/m^2^ (hematological regimen), as well as at low doses (single dose of 1G).^[54,55]^ However, although rituximab has been administered to over 200 IgG4-RD patients worldwide, the optimal dosage and timing of administration remain to be determined, and there have been some similar drawbacks observed in hematology and other autoimmune diseases.^[51]^ For example, 42% of patients treated with rituximab experienced IgG4 RD recurrence, and one-third of patients experienced severe infection or hypogammaglobulinemia in a French nationwide study.

#### 3.6.2. Maintenance of remission

Based on existing retrospective studies and a meta-analysis, rituximab performed better than DMARD therapy in reducing recurrence rate (odds ratio 0.10, 0.01 to 1.63), but the interval between rituximab dosage and dosing regimen varied among patients.^[47,56]^ The maintenance treatment of rituximab is either administered twice every 15 days at a 1 g interval or 4 times a week at 375 mg/m^2^, usually when there are signs of disease onset, rather than at predetermined time intervals. In this regard, a retrospective French multicenter study cohort have shown that regular administration of rituximab at fixed intervals (every 6 months) can prevent IgG4 RD recurrence and has good safety.^[51]^ Another study also provides preliminary evidence that the efficacy of administering 1 g of rituximab every 6 months in maintaining disease remission is the same as the efficacy of administering 1 g of rituximab twice every 15 days.^[57]^

## 4. Conclusions

Here, we report a rare case of IgG4-RD characterized by inflammatory pseudotumor lesions involving the bilateral renal pelvis and adjacent renal parenchyma. In patients with bilateral renal pelvis masses, IgG4-RD should be considered in differential diagnosis to avoid unnecessary surgical intervention, and serum IgG4 level is the important biomarker. Glucocorticoids, immunosuppressive drugs, and biological agents are the first-line therapeutic medication for IgG4-RD.

## Acknowledgments

The authors express their sincere gratitude to the patient for their consent to publish their clinical photographs and radiographs in this case report. The authors also thank the support of Gansu Province Healthcare Industry Research Project (No. GSWSQN2024-11).

## Author contributions

**Conceptualization:** Shaolong Zhang, Hui Ding.

**Data curation:** Shaolong Zhang.

**Formal analysis:** Shaolong Zhang.

**Funding acquisition:** Hui Ding.

**Investigation:** Shaolong Zhang, Hui Ding.

**Methodology:** Shaolong Zhang, Chuanjian Chen.

**Project administration:** Hui Ding.

**Resources:** Shaolong Zhang, Chuanjian Chen.

**Software:** Shaolong Zhang, Chunhao Mo.

**Supervision:** Hui Ding.

**Validation:** Shaolong Zhang, Chuanjian Chen, Chunhao Mo.

**Visualization:** Shaolong Zhang, Chuanjian Chen.

**Writing – original draft:** Shaolong Zhang, Chuanjian Chen, Chunhao Mo.

**Writing – review & editing:** Hui Ding.
